# Reemergence of Clade IIb–Associated Mpox, Germany, July–December 2023

**DOI:** 10.3201/eid3007.240092

**Published:** 2024-07

**Authors:** Patrick E. Obermeier, Clarissa F. Plinke, Annika Brinkmann, Raskit Lachmann, Julia Melchert, Victor M. Corman, Andreas Nitsche, Ulrich Marcus, Axel J. Schmidt, Klaus Jansen, Susanne C. Buder

**Affiliations:** Vaccine Safety Initiative, Berlin, Germany (P.E. Obermeier);; Vivantes Hospital Neukölln, Berlin (C.F. Plinke, S.C. Buder);; Robert Koch-Institute, Berlin (A. Brinkmann, R. Lachmann, A. Nitsche, U. Marcus, K. Jansen);; Charité–Universitätsmedizin Berlin and Berlin Institute of Health, Berlin (J. Melchert, V.M. Corman);; London School of Hygiene and Tropical Medicine, London, UK (A.J. Schmidt)

**Keywords:** Mpox, dermatology, sexually transmitted infections, surveillance, genomic surveillance, epidemiology, vaccination, Germany, viruses, monkeypox virus, MPXV

## Abstract

In July 2023, clade IIb–associated mpox reemerged in Germany at low levels, mainly affecting men who have sex with men. We report a representative case and phylogeny of available genome sequences. Our findings underscore the need for standardized surveillance and indication-based vaccination to limit transmission and help prevent endemicity.

Mpox is caused by infection with monkeypox virus (MPXV). Phylogenomically and clinically, clades I and II can be distinguished; clade II has 2 subclades, IIa and IIb (*1*). In July 2022, the World Health Organization (WHO) declared a global clade IIb–associated mpox outbreak a Public Health Emergency of International Concern (*2*). Worldwide, >87,000 laboratory-confirmed cases were reported by the time the emergency was declared over in May 2023 after a marked decline in case reporting. Overall, the WHO Region of the Americas had the highest number of mpox cases (≈59,000), followed by the European Region (≈26,000), particularly Spain (≈7,600), France (≈4,100), the United Kingdom, and Germany (≈3,700, each). Since 2023, only sporadic cases have been reported across Europe, and no cases were notified in Germany during February–July 2023 (*3*,*4*).

## The Study

**Figure 1:**
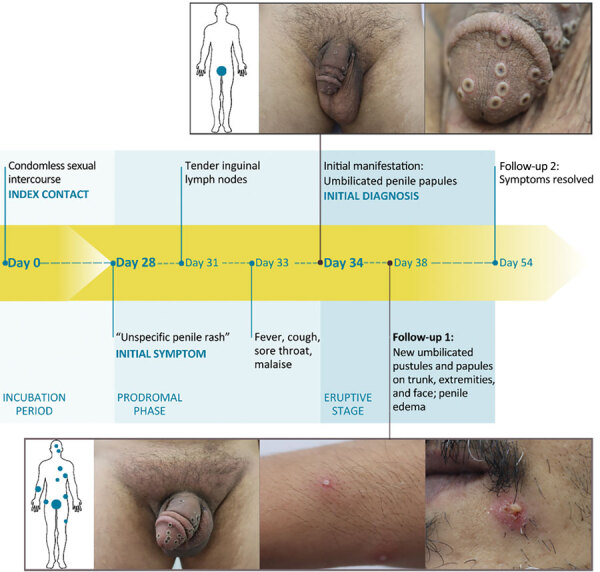
Clinical features and chronology of events in an instructive case from clade IIb–associated reemergence of mpox in Germany, July–December 2023.

In September 2023, a man in his 40s visited a tertiary-care, dermatologic emergency department in Berlin after his general practitioner, a urologist, and a dermatologist could not explain his 1-week history of penile rash, tender lymph nodes, sore throat, cough, malaise, and fever. Signs and symptoms had severely affected his life, as reflected by a Dermatology Life Quality Index (*5*) score of 20 out of 30. Examination revealed the man to be in a reduced general condition, with inguinal lymphadenopathy, a bright red enanthem, and multiple umbilicated penile papules (Figure 1). The man reported a steady female partner but also anal intercourse with an anonymous male partner in a Berlin nightclub 28 days before symptom onset. The man denied any further sexual contact since that encounter and confirmed no mpox or smallpox vaccination nor recent travels abroad. 

Testing revealed MPXV-positive results (LightMix Modular Monkeypox Virus; TIB MOLBIOL, https://www.tib-molbiol.de) obtained by penile papule swab sample (cycle threshold value 27.47) and by urine sample (cycle threshold value 35.34). The patient received antipyretic and analgetic medications. Symptoms resolved, and MPXV PCR results were negative 26 days after disease onset. Further tests revealed negative results for hepatitis B and C viruses, HIV, *Chlamydia trachomatis*, *Mycoplasma genitalium*, *Neisseria gonorrhoeae*, and *Treponema pallidum*.

**Figure 2:**
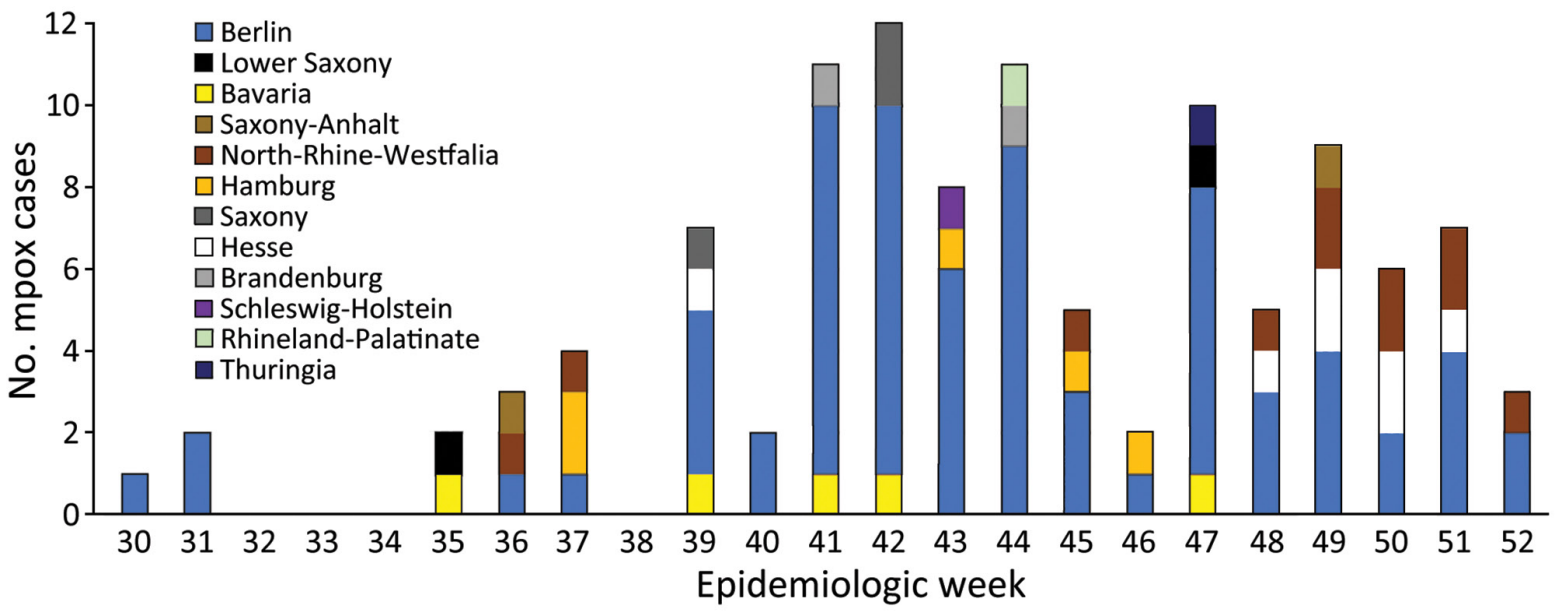
Number of clade II–associated mpox cases in Germany, July–December 2023, by federal state and reporting week (weeks 30**–**52). Total number of cases was 110.

We considered the case-patient significant because few mpox cases were being reported in Germany at the time and he had been misdiagnosed by 3 different healthcare experts despite typical manifestations. An examination of national surveillance data revealed 110 laboratory-confirmed mpox cases during July–December 2023, after 25 weeks without case reports (*4*). Men accounted for all but 1 patient; median age was 36 years (range 17**–**80 years). Reported cases originated from 12 of 16 federal states, but most notifications came from the federal state of Berlin (62/110) (Figure 2). Of the 90 cases with information on the probable route of transmission, 89 were men who have sex with men. Most cases with information on the place of infection (n = 88) traced acquisition to Berlin (59/88), and 10 cases were imported (2 cases each from the United States, Spain, and the Netherlands and 1 case each from Egypt, Greece, Saudi Arabia, and Thailand).

Investigators performed a phylogenetic analysis of 29 available sequences from the reemergence of mpox in Germany, generated by using native Illumina NextSeq (Illumina, https://www.illumina.com) or amplicon-based Oxford Nanopore MinION (Oxford Nanopore Technologies, https://nanoporetech.com) sequencing (A. Brinkmann et al., unpub data, https://doi.org/10.1101/2022.10.20.512862). We used those sequences and a selection of 58 publicly available MPXV sequences from GISAID (https://www.gisaid.org) for an alignment of the central conserved region (≙vaccinia virus genes F9L-A24R) by using Mafft version 7.307 with default parameters (https://mafft.cbrc.jp). We performed phylogenetic analysis by using IQ-TREE v1.6.12 (http://www.iqtree.org) with the maximum-likelihood method, general time reversible plus gamma distribution plus invariate sites model, and 1,000 bootstrap replicates, which showed that all available Germany genome sequences belonged to clade IIb. Most genome sequences (n = 17) were B.1 lineage sequences, followed by C.1 (n = 10) and B.1.20 (n = 2). The MPXV genome of the case-patient we describe (GenBank accession no. PP002089) could be assigned to lineage B.1 (Appendix Figure 1), with highest identity to other genomes from Berlin during October 2023. A second cluster of sequences from Berlin, separated by >10 mutations, circulated simultaneously. Three C.1 sequences from Berlin showed 100% identity, and the sequences were also identical to a genome from Portugal. However, C.1 strains from Berlin also clustered with genomes from China in July 2023. Three C.1 genomes from Berlin showed 3 unique mutations to cocirculating C.1 genomes from China, Japan, and Portugal.

We ascertained information on mpox vaccination from available data for 96 of 110 cases: 54 case-patients (56%) were vaccinated, and 30 had received 2 doses, 16 received 1 dose, and 8 received an undisclosed number of doses. In Germany, >47,537 persons were vaccinated with >1 dose of mpox vaccine since its approval in July 2022 through the end of November 2023, according to the framework of voluntary national vaccination monitoring (*6*). On the basis of sexual behavior data from representative population surveys in Germany (*7*) and the European MSM Internet Survey (EMIS-2017) (*8*), we estimated the denominator of the German population likely to be at high risk for mpox—that is, self-identified gay men reporting >5 anal intercourse partners within the previous 12 months—to be ≈90,000 persons (*9*,*10*) (Appendix Figure 2). When adding the reported number of mpox cases and the reported number of vaccinations for the numerator, we assumed a maximum proportion of 58% of self-identified gay men at high risk for mpox to be immunized.

## Conclusions

We report re-emergence of mpox in Germany, mainly in Berlin, since July 2023, after 6 months without a single case in the German notification system. Transmission remained at a relatively stable, low level until December 2023 and mainly affected men who have sex with men. A thorough, sensitive assessment of a patient’s sexual history remains essential in the diagnostic workup, and a reported long incubation period of >21 days (*11*) does not rule out mpox. Prior vaccination also does not automatically exclude mpox.

The phylogenomic analysis supports low-level transmission of clade IIb MPXV in Germany, particularly in Berlin, and intra-lineage diversity indicates >1 origin. Genome sequence similarity among strains from several countries in Europe, the Americas, and Asia underscores the status of mpox as an international concern. Our investigation supports the need for genomic surveillance to monitor transmission and the circulation and cocirculation of lineages and clades, particularly because of the increase of clade I–associated mpox in Africa (*12*,*13*).

We have based this study on data from national passive surveillance and our data likely underestimates the true number of case-patients because of such factors as ascertainment bias (as was the case with our case-patient) and omission of paucisymptomatic or clinically inapparent infections. We also concede that it is not possible to understand all transmission chains, given the often incomplete data in the notification system. We would require additional study data to assess disease severity or vaccine effectiveness.

The persistence of MPXV transmission worldwide, combined with vaccination monitoring and surveillance data, indicates that herd immunity has not yet been achieved, particularly in the indication group. Of note, official surveillance data offer an unclear picture of whether all vaccinated persons actually met the criteria for vaccination (or the narrower definition used for the denominator calculation in our study). Persons who were not, strictly speaking, at risk for mpox might have been vaccinated, and some persons at risk likely did not get vaccinated.

Our study elucidates the need for increased awareness of mpox among healthcare professionals and at-risk persons to reduce further spread and endemicity. We advocate indication-based vaccination, including healthcare professionals at risk, and improved vaccine communication and education to increase vaccine uptake as a public health measure.

Appendix Additional information on reemergence of clade IIb–associated mpox, Germany, July–December 2023.

## References

[1] Zhu J, Yu J, Qin H, Chen X, Wu C, Hong X, et al. Exploring the key genomic variation in monkeypox virus during the 2022 outbreak. BMC Genom Data. 2023;24:67. 10.1186/s12863-023-01171-0 37968621 PMC10652487

[2] Wenham C, Eccleston-Turner M. Monkeypox as a PHEIC: implications for global health governance. Lancet. 2022;400:2169–71. 10.1016/S0140-6736(22)01437-4 35926551 PMC9342918

[3] World Health Organization. Multi-country outbreak of mpox, External situation report #22—11 May 2023 [cited 2024 Jan 7]. https://www.who.int/publications/m/item/multi-country-outbreak-of-mpox--external-situation-report--22---11-may-2023

[4] Robert Koch-Institut. SurvStat@RKI 2.0 [cited 2023 Nov 11] https://survstat.rki.de

[5] Finlay AY, Khan GK. Dermatology Life Quality Index (DLQI)—a simple practical measure for routine clinical use. Clin Exp Dermatol. 1994;19:210–6. 10.1111/j.1365-2230.1994.tb01167.x 8033378

[6] Robert Koch-Institut. Mpox vaccination monitoring June 2022–December 2023 [cited 2023 Dec 22]. https://www.rki.de/DE/Content/Infekt/Impfen/ImpfungenAZ/Affenpocken/Affenpocken-Impfmonitoring.pdf

[7] Von Rüden U. AIDS in the public consciousness of the Federal Republic of Germany 2016: knowledge, attitudes and behaviour for protection against HIV/AIDS and other sexually transmitted infections (STIs). BZgA research report. Cologne: Federal Centre for Health. Aufklarung (Hambg). 2017 [cited 2024 Apr 23]. https://www.bzga.de/fileadmin/user_upload/PDF/studien/aioeb_2016_kurzbericht--a344710f2ec9af0c39b1d0bfe2ce140d.pdf

[8] Weatherburn P, Hickson F, Reid DS, Marcus U, Schmidt AJ. European men-who-have-sex-with-men internet survey (EMIS-2017): design and methods. Sex Res Soc Policy. 2020;17:543–57. 10.1007/s13178-019-00413-0

[9] Koch J, Vygen-Bonnet S, Bogdan C, Burchard G, Garbe E, Heininger U, et al. Scientific justification of the STIKO for the recommendation to vaccinate against monkeypox with *Imvanex* (MVA-Impfstoff). Epidemiologisches Bulletin: Current Data and Information on Infectious Diseases and Public Health. 2022;25-26:5–17 [cited 2023 Nov 26]. https://edoc.rki.de/bitstream/handle/176904/9878/EB-25-26-2022-Begr%c3%bcndung-Affenpocken.pdf

[10] Standing Committee on Vaccination (STIKO). Recommendations of the Standing Committee on Vaccination (STIKO) at the Robert Koch Institute–2023. Epidemiologisches Bulletin: Current Data and Information on Infectious Diseases and Public Health. 2023;4:3–68 [cited 2024 Apr 27] https://www.rki.de/EN/Content/infections/Vaccination/recommandations/04_23_englisch.pdf

[11] McFarland SE, Marcus U, Hemmers L, Miura F, Iñigo Martínez J, Martínez FM, et al. Estimated incubation period distributions of mpox using cases from two international European festivals and outbreaks in a club in Berlin, May to June 2022. Euro Surveill. 2023;28:2200806. 10.2807/1560-7917.ES.2023.28.27.2200806 37410383 PMC10370040

[12] Kibungu EM, Vakaniaki EH, Kinganda-Lusamaki E, Kalonji-Mukendi T, Pukuta E, Hoff NA, et al.; International Mpox Research Consortium. Clade I–associated mpox cases associated with sexual contact, the Democratic Republic of the Congo. Emerg Infect Dis. 2024;30:172–6. 10.3201/eid3001.231164 38019211 PMC10756366

[13] European Centre for Disease Prevention and Control. Implications of the EU/EEA of the outbreak of mpox caused by monkeypox virus clade I in the Democratic Republic of the Congo. Stockholm: European Centre for Disease Prevention and Control; 2023 [cited 2024 Apr 27]. https://www.ecdc.europa.eu/sites/default/files/documents/Implications-mpox-drc-TAB-erratum.pdf

