# Supplementary material for: Reemergence of Clade IIb–Associated Mpox, Germany, July–December 2023
Source: Emerg Infect Dis. 2024 Jul;30(7):1416–9. doi: 10.3201/eid3007.240092 (PMC11210634; doi:10.3201/eid3007.240092)
Supplement: Appendix — Additional information on reemergence of clade IIb–associated mpox, Germany, July–December 2023. [file 24-0092-Techapp-s1.pdf]

Article DOI: http://doi.org/10.3201/eid3007.240092

EID cannot ensure accessibility for supplementary materials supplied by authors. Readers who have difficulty accessing supplementary content should contact the authors for assistance.

Reemergence of Clade IIb–Associated Mpox in Germany, July–December 2023

Appendix

Data Availability

Sequence data generated in this study have been deposited in GenBank and are publicly available:

- OR449306
- OR449307
- OR519724
- OR519723
- OR743493
- OR743494
- OR743495
- OR743496
- OR743497
- OR743498
- OR743499
- OR743500
- OR777670
- OR777671
- OR777672

- OR777673
- OR777674
- OR777675
- OR777676
- OR777677
- OR777678
- OR777679
- OR777680
- OR777681
- PP002088
- PP002089
- PP002090
- PP002091
- PP002092

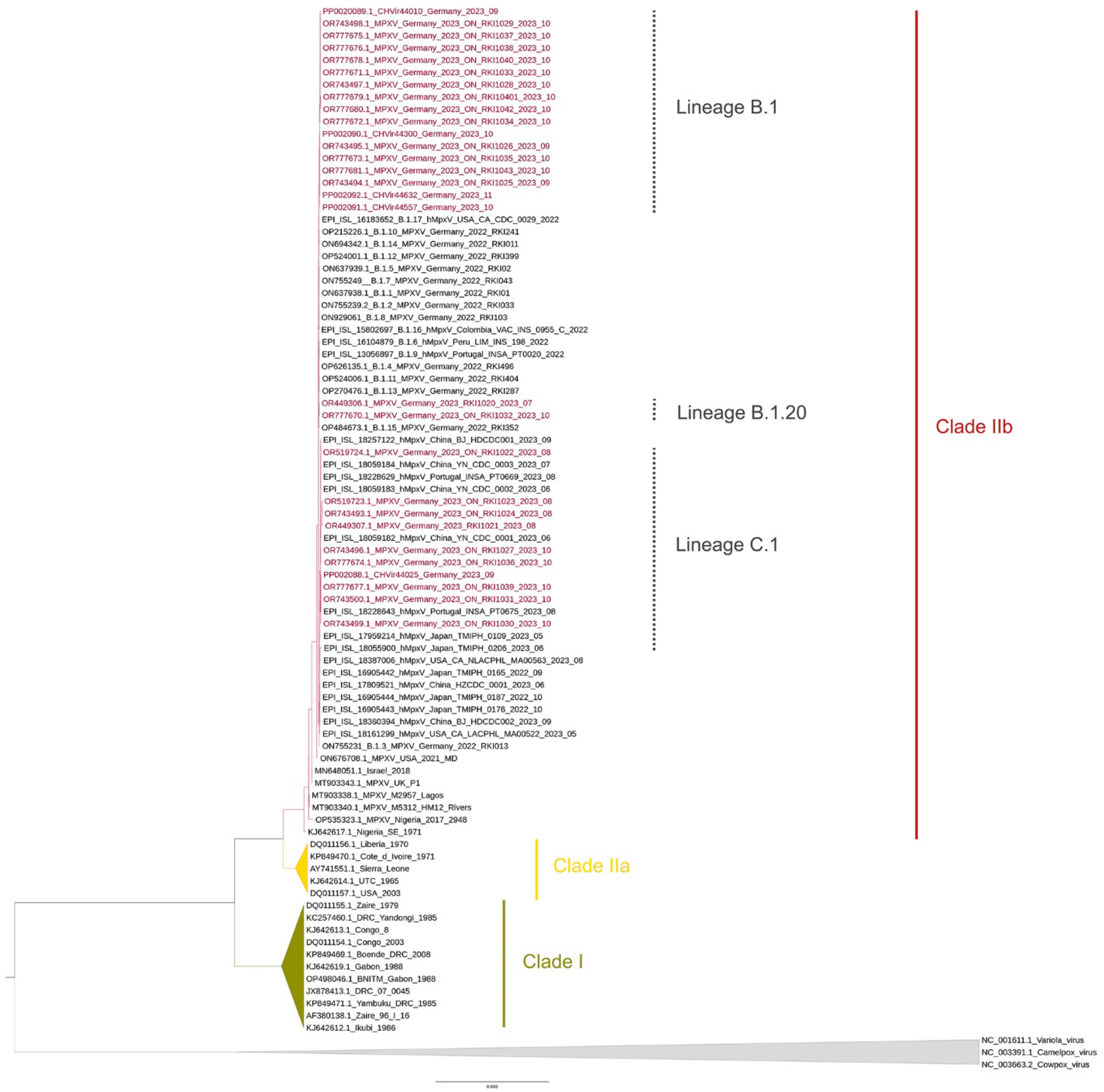

Appendix Figure 1. Phylogenetic tree of mpox virus nucleotide genome sequences from the clade IIb-associated re-emergence of mpox in Germany, July–December 2023. Red font color indicates sequences (n = 29) that were generated for this study.

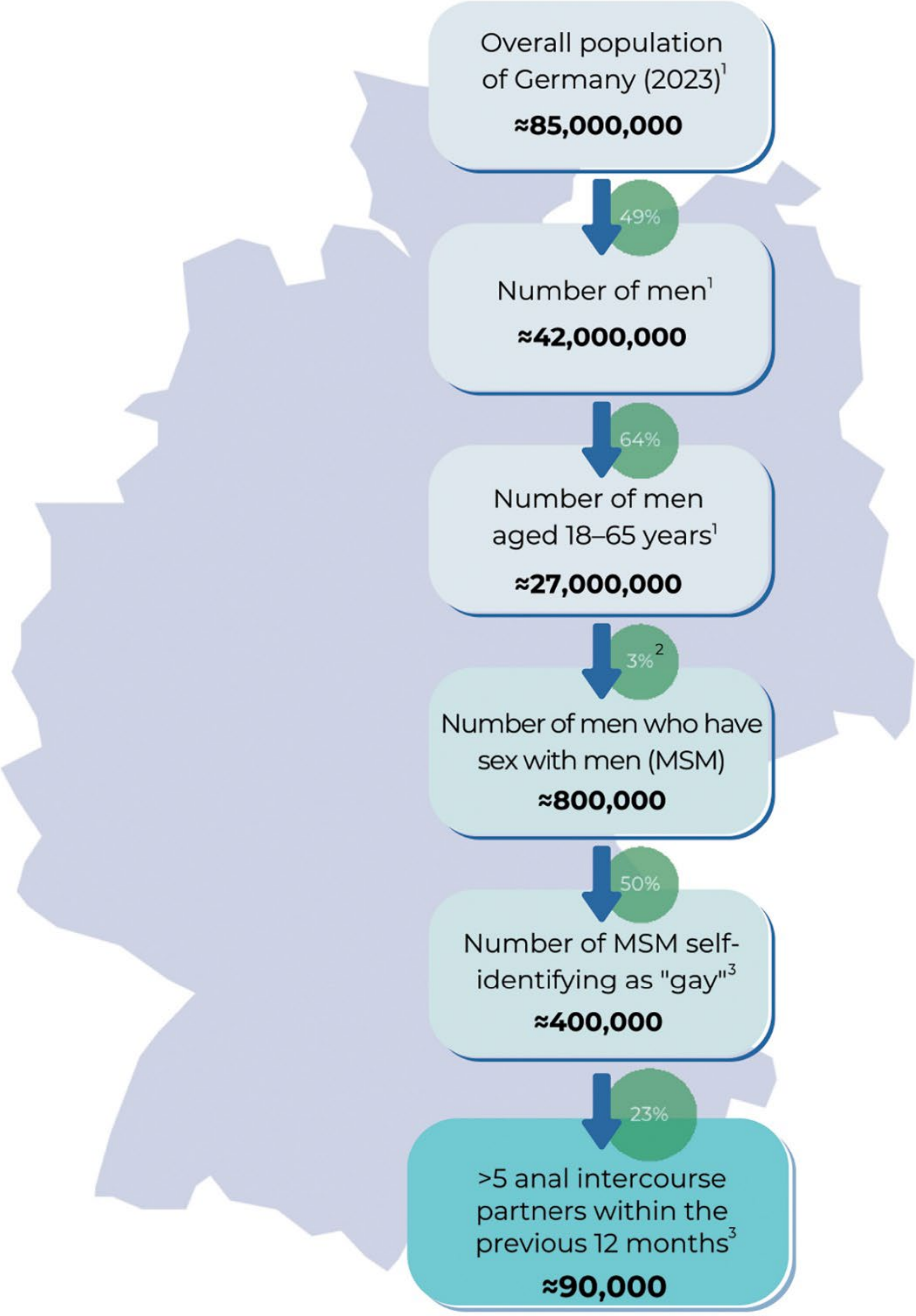

Appendix Figure 2. Flowchart relevant to the re-emergence of clade IIb-associated mpox in Germany, July–December 2023, estimating the denominator of persons at risk in Germany.

References

1. Von Rüden U. AIDS in the public consciousness of the Federal Republic of Germany 2016: knowledge, attitudes and behaviour for protection against HIV/AIDS and other sexually transmitted infections (STIs). BZgA research report. Cologne: Federal Centre for Health. Aufklarung (Hambg). 2017 [cited 2024 Apr 23]. https://www.bzga.de/fileadmin/user_upload/PDF/studien/aioeb_2016_kurzbericht--a344710f2ec9af0c39b1d0bfe2ce140d.pdf

2. Weatherburn P, Hickson F, Reid DS, Marcus U, Schmidt AJ. European men-who-have-sex-with-men internet survey (EMIS-2017): design and methods. Sex Res Soc Policy. 2020;17:543–57. https://doi.org/10.1007/s13178-019-00413-0

3. Federal Statistical Office DESTATIS. Population. 2024 [cited 2024 Apr 28]. https://www.destatis.de/DE/Themen/Gesellschaft-Umwelt/Bevoelkerung/Bevoelkerungsstand/_inhalt.html
